# Pre- and Early Post-treatment With *Arthrospira platensis* (Spirulina) Extract Impedes Lipopolysaccharide-triggered Neuroinflammation in Microglia

**DOI:** 10.3389/fphar.2021.724993

**Published:** 2021-09-09

**Authors:** Anna Piovan, Jessica Battaglia, Raffaella Filippini, Vanessa Dalla Costa, Laura Facci, Carla Argentini, Andrea Pagetta, Pietro Giusti, Morena Zusso

**Affiliations:** Department of Pharmaceutical and Pharmacological Sciences, University of Padua, Padua, Italy

**Keywords:** neuroinflammation, microglia, spirulina, pre-treatment, post-treatment, pro-inflammatory cytokines

## Abstract

**Background:** Uncontrolled neuroinflammation and microglia activation lead to cellular and tissue damage contributing to neurodegenerative and neurological disorders. Spirulina (*Arthrospira platensis* (Nordstedt) Gomont, or *Spirulina platensis*), a blue-green microalga, which belongs to the class of cyanobacteria, has been studied for its numerous health benefits, which include anti-inflammatory properties, among others. Furthermore, *in vivo* studies have highlighted neuroprotective effects of Spirulina from neuroinflammatory insults in different brain areas. However, the mechanisms underlying the anti-inflammatory effect of the microalga are not completely understood. In this study we examined the effect of pre- and post-treatment with an acetone extract of Spirulina (E1) in an *in vitro* model of LPS-induced microglia activation.

**Methods:** The effect of E1 on the release of IL-1β and TNF-α, expression of iNOS, nuclear factor erythroid 2–related factor 2 (Nrf2), and heme oxygenase-1 (HO-1), and the activation of NF-κB was investigated in primary microglia by ELISA, real-time PCR, and immunofluorescence.

**Results:** Pre- and early post-treatment with non-cytotoxic concentrations of E1 down-regulated the release of IL-1β and TNF-α, and the over-expression of iNOS induced by LPS. E1 also significantly blocked the LPS-induced nuclear translocation of NF-κB p65 subunit, and upregulated gene and protein levels of Nrf2, as well as gene expression of HO-1.

**Conclusions:** These results indicate that the extract of Spirulina can be useful in the control of microglia activation and neuroinflammatory processes. This evidence can support future *in vivo* studies to test pre- and post-treatment effects of the acetone extract from Spirulina.

## Introduction

Neuroinflammation is a complex and multifactorial response of the central nervous system (CNS) directed at protecting the brain from endogenous and exogenous noxious stimuli and restoring tissue homeostasis and integrity. Although the protective purpose of the inflammatory response, a minimal unbalance in its components may result harmful and contribute to virtually every neurodegenerative/neurological disorder (Amor et al., 2014; Sochocka et al., 2017; Skaper et al., 2018). Microglia are the immunocompetent cells in the CNS that largely participate in neuroinflammatory processes (Gomez-Nicola and Perry, 2015; Colonna and Butovsky, 2017). Once activated these cells secrete pro-inflammatory cytokines, chemokines, nitric oxide, and oxygen radicals, that contribute to the development of CNS damage (Lucas et al., 2006). Microglia activation mechanisms begin with the recognition of pathogens through a limited number of pattern-recognition receptors, including Toll-like receptors (TLRs), which are involved in the initiation of innate immune response (Bsibsi et al., 2002; Kawai and Akira, 2010). Among TLR family members, TLR2, 4, and 9 are mainly implicated in neurodegenerative diseases such as Alzheimer’s disease, Parkinson’s disease, and amyotrophic lateral sclerosis (Fiebich et al., 2018). TLR activation initiates diverse signal transduction pathways, including the activation of the transcription factor nuclear factor (NF)-κB, which regulates the transcription of many genes involved in immunity and inflammation (Bryant et al., 2010). Therefore, microglia activation and TLRs are regarded as potential targets for therapeutic intervention in brain diseases associated with neuroinflammation (Liu et al., 2019).

In recent years, microalgae are gaining a high interest for their nutritional and therapeutic applications being important sources of food ingredients and bioactive products with numerous health benefits (Gómez-Zorita et al., 2019). Microalgae are prokaryotic or eukaryotic single-cell organisms, found in fresh water and marine systems. They produce half of the atmospheric oxygen and a variety of compounds, such as photosynthetic pigments, polyunsaturated fatty acids, vitamins, minerals, fibers, polysaccharides, enzymes, and peptides (Khan et al., 2018; Galasso et al., 2019). Among the microalgae, Spirulina (*Arthrospira platensis* (Nordstedt) Gomont, or *Spirulina platensis*) is a photosynthetic, filamentous, spiral shaped, and blue-green cyanobacterium that has a long history as dietary supplement, based on its high content of proteins (60–70% of the microalga dry weight). It also contains vitamins (in particular vitamin B12), minerals (iron, calcium, magnesium, zinc, manganese, phosphorus, and potassium), essential fatty acids (e.g., γ-linoleic acid, palmitic acid, linoleic acid, oleic acid), polysaccharides, glycolipids and sulfolipids, enzymes (e.g., superoxide dismutase), and various pigments, including phycocyanins, chlorophylls, and carotenoids (Colla et al., 2004; Niccolai et al., 2019). Recently, Spirulina has been widely studied for its numerous health benefits, which include antibacterial, antiviral, antioxidant, and anti-inflammatory properties (Remirez et al., 2002; Kulshreshtha et al., 2008; Mallikarjun Gouda et al., 2015; Wu et al., 2016; Finamore et al., 2017). Furthermore, many studies have evidenced the neuroprotective properties of Spirulina in multiple models of CNS diseases, such as Parkinson’s disease, schizophrenia, ischemic brain damage and in lipopolysaccharide (LPS)-induced neuroinflammation (Strömberg et al., 2005; Chen et al., 2012; Pabon et al., 2012; Lima et al., 2017; Haider et al., 2021). However, the protective properties of Spirulina have been shown mainly after a pre-treatment (i.e., prophylactic effect), whereas its effect after inflammatory stimuli has been only partially investigated.

In the present study we examined the effects of pre- and post-treatment with an acetonic extract of the microalga Spirulina (E1) on LPS-induced microglial activation *in vitro*. Then, potential mechanisms that regulate the observed effects were also clarified. We found that pre-treatment and early post-treatment with E1 lowered microglia activation by inhibiting the release of pro-inflammatory cytokines and gene expression of inflammatory markers through mechanisms that involve NF-κB and the nuclear factor erythroid 2–related factor 2 (Nrf2).

## Material and Methods

### Reagents

Unless otherwise specified, all reagents were from Sigma-Aldrich (Milan, Italy). Tissue culture media, antibiotics, and fetal bovine serum (FBS) were obtained from Life Technologies (San Giuliano Milanese, Italy). LPS (Ultra-Pure LPS-EB from *Escherichia coli*, 0111:B4 strain) was purchased from InvivoGen (InvivoGen Europe, Toulouse, France). Dried Spirulina was purchased from a local health-food store. Primary antibodies included: mouse anti-p65 (NF-κB p65, Santa Cruz Biotechnology, Santa Cruz, CA, United States, Cat. sc-8008), mouse anti-iNOS (NOS2, Santa Cruz Biotechnology, Santa Cruz, CA, United States, Cat. sc-7271), and rabbit anti-Nrf2 (GeneTex Inc., Irvine, CA, United States, Cat. GTX103322). Alexa Fluor 488 and 555 secondary antibodies were from and Invitrogen (Milan, Italy, Cat. A11008 and A21422, respectively). Enzyme-linked immunosorbent assay (ELISA) kits were obtained from Antigenix America (Huntington Station, NY, United States). Falcon tissue culture plasticwares were purchased from BD Biosciences (SACCO srl, Cadorago (CO), Italy).

### Preparation and Analysis of Spirulina Extract

Properly hydrated powder of Spirulina was ground in a mortar and extracted with acetone in an ultrasonic bath for 20 min at room temperature and then centrifuged at 4,400 rpm for 10 min at 4°C. The extraction process was repeated three times and the combined extracts were concentrated under reduced pressure in a rotary evaporator. The concentrated solution was then lyophilized. Finally, the extract was stored at 4°C until use.

Chlorophyll a, chlorophyll b, and total carotenoid content was determined as described by Yang et al. (1998). Pheophytin content was measured according to the method of Lichtenthaler (1987). One aliquot of the extract was solubilized with acetone:water (4:1) and, after appropriate dilution, the maximum absorbance was read at 663, 646, 470, 653, and 665 nm for chlorophyll a, chlorophyll b, carotenoids, pheophytin a, and pheophytin b, respectively. The content of pigments was calculated using the following equations:

$$\mathrm{Chlorophyll}\;a\left(\mathrm{\mu g}/\mathrm{mL}\right)=12{\text{.}25\text{A}}_{663}-2{\text{.}25\text{A}}_{646}$$

$$\mathrm{Chlorophyll}\;\text{b}\left(\mathrm{\mu g}/\mathrm{mL}\right)=20{\text{.}31\text{A}}_{646}-2{\text{.}25\text{A}}_{663}$$

$$\mathrm{Total}\;\mathrm{carotenoids}\left(\mathrm{\mu g}/\mathrm{mL}\right)=\left(\mathrm{1000}A_{\mathrm{470}}-2.27\mathrm{Chlorophyll a-81}\mathrm{.4 Chlorophyll}\;b\right)/227$$

$$\text{Pheophytin}\;a\left(\mathrm{\mu g}/\mathrm{mL}\right)\mathrm{=2}2{\text{.}42\text{A}}_{665}\mathrm{- 6}{\text{.}81\text{A}}_{653}$$

$$\text{Pheophytin}\;\text{b}\left(\mathrm{\mu g}/\mathrm{mL}\right)\mathrm{=40}{\text{.}17\text{A}}_{653}\mathrm{- 18}{\text{.}58\text{A}}_{665}$$

Results were expressed as mg/g of dry weight of extract.

The carotenoid analysis was performed using an Agilent 1100 HPLC Series System (Agilent, Santa Clara, CA, United States) equipped with degasser, quaternary gradient pump, column thermostat, and UV-Vis detector. A Gemini 5-µm C6-Phenyl column (250 × 4.6 mm) from Phenomenex (Torrance, CA, United States) was employed, at 40°C. Analyses were done in the isocratic mode, using acetonitrile:methanol (10:90; v/v) at a flow rate of 1 ml min^−1^, with an injection volume of 10 μl; detection was at 280, 365, and 460 nm. Carotenoid content was expressed as β-carotene equivalents.

### Cell Cultures

Animal-related procedures were performed in accordance with EU Directive (2010/63/EU) for animal experiments and those of the Italian Ministry of Health (D.L. 26/2014) and were approved by the Institutional Review Board for Animal Research (Organismo Preposto al Benessere Animale, OPBA) of the University of Padua and by the Italian Ministry of Health (protocol number 41451.N.N8P). Animals were maintained under controlled conditions (22–24°C, 50%–60% humidity), with free access to water and food on a 12 h light/dark cycle (lights on at 7:00 am). One-day-old Sprague-Dawley rat pups (CD strain) of both sexes were rapidly decapitated, minimizing suffering, discomfort, or stress. Primary microglial cells were isolated from mixed glial cell cultures prepared from the cerebral cortex, as previously described (Skaper et al., 2012). When mixed glial cultures reached confluence (typically 7 days after isolation), microglia were separated from the astroglial monolayer by shaking (200 rpm for 1 h at 37°C), re-suspended in high-glucose Dulbecco’s modified eagle medium (DMEM) supplemented with 2 mM L-glutamine, 10% heat-inactivated FBS, 100 units/ml penicillin, 100 μg/ml streptomycin and 50 μg/ml gentamicin (growth medium), and plated on poly-L-lysine-coated (10 μg/ml) plastic wells at a density of 1.50 × 10^5^ cells/cm^2^. Cells were allowed to adhere for 45 min and then washed to remove non-adhering cells. Cultures obtained using the shaking procedure generated 97% microglia immunopositive to a primary polyclonal antibody against ionized calcium binding adaptor molecule 1 (Iba1, 1:800, Wako Chemicals United States Inc., Richmond, VA, United States, Cat. 019–19741), a marker for microglia cell types. Cells were maintained at 37°C in a humidified atmosphere containing 5% CO2/95% air. LPS was suspended in endotoxin-free water (InvivoGen). E1 was suspended in dimethylsulfoxide (DMSO) just before use and added to the cultures so as not to exceed 0.1% of the total volume. Control cultures contained the same concentration of DMSO.

### Cell Treatment

Cells were seeded in poly-L-lysine coated 96-well plates (50,000 cells/well) in growth medium and allowed to adhere overnight. In the pre-treatment experiments, serum-containing medium was replaced with serum-free medium 2 h before pre-treatment with E1 for 1 h, followed by stimulation with 100 ng/ml Ultra-Pure LPS-EB. In the post-treatment experiments, 2 h after serum starvation, microglia were stimulated with 100 ng/ml LPS and 2 or 4 h later cells were treated with E1. In both conditions, microglia were stimulated with LPS for 6 or 16 h for the evaluation of gene expression or cytokine release, respectively.

### Cell Viability Assay

Microglial cell viability was evaluated by a colorimetric method utilizing the protein-binding dye sulforhodamine B (SRB) (Skehan et al., 1990; Piovan et al., 2021). Growth medium was replaced with serum-free medium 2 h before treatment with increasing concentrations of E1 for 16 h. After the incubation, the medium was replaced with cold 10% trichloroacetic acid, and plates were incubated for 1 h at 4°C. Following this fixation step, cells were stained with 0.4% SRB and left at room temperature for 30 min. The bound protein stain was solubilized with 10 mM Tris base. The absorbance was then measured at 570 nm in a microplate reader. Absorbance of vehicle-treated cultures was considered as 100% cell viability.

### Cytokine Determination

After treatments, culture media were collected and IL-1β and TNF-α assayed using commercially available ELISA kits, according to the manufacturer’s instructions. Cytokine concentrations (pg/ml) in the medium were determined by reference to standard curves obtained with known amounts of IL-1β or TNF-α and the results expressed as percentage relative to corresponding control value.

### Real-Time Polymerase Chain Reaction

At the end of 6-h LPS stimulation, total RNA was extracted from cells by QIAzol (Invitrogen), according to the manufacturer’s instructions. RNA integrity and quantity were determined by RNA 6000 Nano assay in an Agilent BioAnalyser (Thermo Scientific, Milan, Italy). Reverse transcription was performed with Superscript III reverse transcriptase (Invitrogen). The real-time PCR reaction was performed as described previously (Barbierato et al., 2017). Primer sequences are listed in Table 1. Amounts of each gene product were calculated using linear regression analysis from standard curves, demonstrating amplification efficiencies ranging from 90 to 100%. Dissociation curves were generated for each primer pair, showing single product amplification. Data are presented as specific ratio between the gene of interest and the reference gene (β-actin).

**TABLE 1:** Primers for real-time PCR used in this study.

Gene target	Primer name	Sequence (5′-3′)
INOS	iNOS For	GGG​AAC​ACC​TGG​GGA​TTT​TC
	iNOS Rev	CAC​AGT​TTG​GTC​TGG​CGA​AG
Nrf2	Nrf2 For	GGA​TAT​TCC​CAG​CCA​CGT​TGA
	Nrf2 Rev	AAT​CAG​TCA​TGG​CCG​TCT​CC
HO-1	HO-1 For	GTT​TCC​TGT​TGG​CGA​CCG​TG
	HO-1 Rev	GCC​AGG​CAA​GAT​TCT​CCC​CT
β-actin	β-actin For	GAT​CAG​CAA​GCA​GGA​GTA​CGA​TGA
	β-actin Rev	GGT​GTA​AAA​CGC​AGC​TCA​GTA​ACA

### Immunofluorescence and Image Analysis

Microglia were grown on coverslips in 24-well plates, pre-treated for 1 h with E1 before stimulation with 100 ng/ml Ultra-Pure LPS-EB for an additional 90 min for the analysis of NF-κB activation, or 16 h for the analysis of iNOS and Nrf2 expression. Cells were fixed with 4% paraformaldehyde (pH 7.4, for 15 min at room temperature) and subsequently non-specific staining was blocked by incubating with 5% normal goat serum/0.1% Triton X-100 in PBS for 1 h at room temperature. Cells were then incubated sequentially with primary (2 h) and secondary antibody (1 h) in the above blocking solution. The antibodies used were mouse anti-p65 (NF-κB p65, 1:500) primary antibody, mouse anti-iNOS (1:500), and rabbit anti-Nrf2 (1:200) followed by Alexa Fluor 488- or 555-conjugated secondary antibodies (1:1000). Cells were thoroughly washed between steps with PBS. Immunostaining control included omission of the primary antibody. Nuclei were stained with 4,6-diamidino-2-phenylindole (DAPI; 0.1 μg/ml) and coverslips were mounted on microscope slides with Fluoromount-G mounting medium (Fisher Scientific, Milan, Italy) (Zusso et al., 2017). Fluorescent images were captured with a confocal laser-scanning microscope (Zeiss LSM 800; Carl Zeiss AG, Germany) and microscope settings were kept constant for all images. For each image, three z-stacks (50 μm optical section, 1.5 μm total Z-span) were acquired with a 63x, NA 1.4, oil-immersion objective. All images were taken considering the middle of nuclei as the central plane for z-stack. ImageJ software (National Institutes of Health, Bethesda, MD, United States) was used to flatten each z-stack image into a single image, representing the sum of the contributes from each focal plane. NF-κB p65 fluorescence emission intensity of single cells was profiled using ImageJ software. To quantitatively evaluate subcellular distribution of the p65 subunit, the relative staining intensities in the nucleus and cytoplasm were monitored from five random fields for each condition from three independent experiments. Cytoplasmic and nuclear fluorescence intensities were calculated using ImageJ software and are expressed as a percentage of nuclear and cytoplasmic staining.

### Statistical Analysis

All data represent the results of at least three independent experiments. Data were analyzed using GraphPad Prism Software, version 6.0 (GraphPad Software, Inc., San Diego, CA, United States). Results are expressed as mean ± SEM. Statistical analyses to determine group differences were performed either by two-sample equal variance Student’s *t* test, or by one-way analysis of variance (ANOVA) followed by Sidak’s *post hoc* test for multiple comparisons. A value of *p* < 0.05 was considered to indicate statistically significant differences. Additional details are provided in the figure legends, where appropriate.

## Results

### Analysis of Spirulina Extract

**FIGURE 1:**
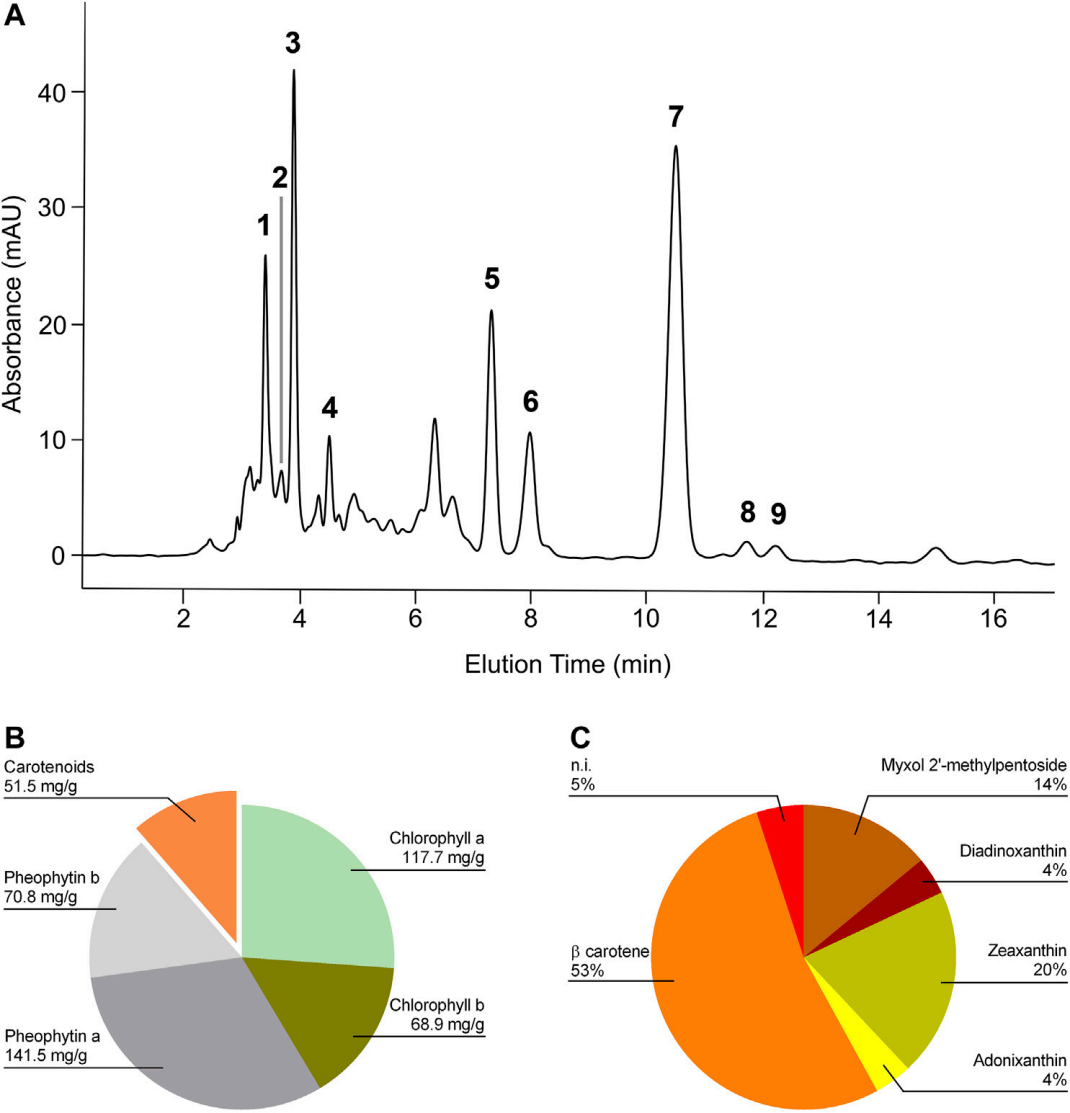
HPLC profile of the Spirulina acetone extract and its relative composition. **(A)** Chromatogram was registered at 460 nm. Retention times: myxol 2′-methyl pentoside, 3.45 min (1); diadinoxanthin, 3.72 min (2); zeaxanthin, 3.94 min (3); adonixanthin, 4.5 min (4); unidentified xanthophylls, 3.18, 4.36, and 4.98 min; chlorophyll a, 7.35 min (5); chlorophyll b, 8.01 min (6); β carotene, 10.5 min (7); pheophytin a, 11.76 min (8); pheophytin b, 12.25 min (9). **(B)** Content of chlorophyll a, chlorophyll b, pheophytin a, pheophytin b, and total carotenoids expressed as mg/g of dry extract. **(C)** Relative carotenoid content in E1. β-carotene represented 53% of total carotenoids present.

HPLC UV-Vis analysis of E1 led to the identification of β-carotene, several xanthophylls, chlorophylls, and pheophytins (Figure 1A). Figure 1B shows the content of chlorophyll a, chlorophyll b, pheophytin a, pheophytin b, and total carotenoids expressed as mg/g of dry extract. β-carotene represented 53% of total carotenoids present and among xanthophylls, zeaxanthin and diadinoxanthin were the most abundant (Figure 1C).

### Effect of Pre-treatment With Spirulina Extract on Microglia Inflammatory Response

**FIGURE 2:**
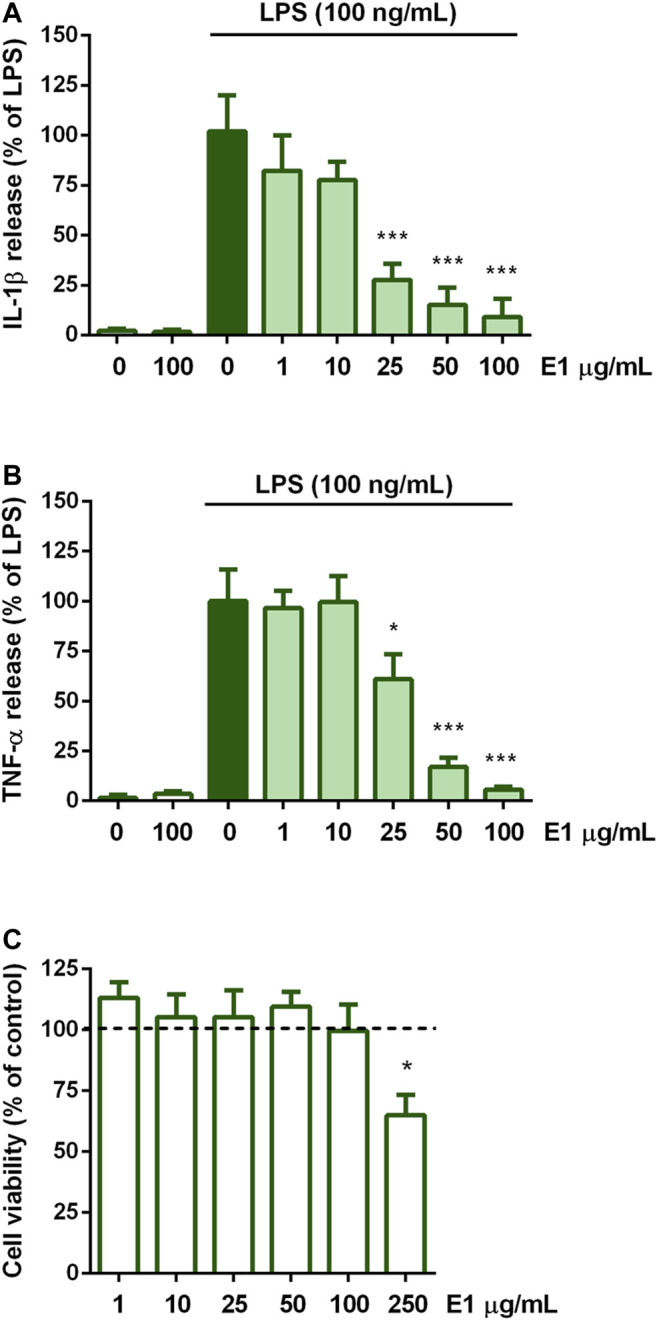
Effect of pre-treatment with Spirulina extract on cytokine release from LPS-stimulated cortical microglia and cell viability analysis. Microglia were cultured for 24 h in 10% serum-containing medium, which was replaced with serum-free medium before pre-treatment with E1 (1–100 μg/ml) for 1 h and further stimulation with 100 ng/ml LPS for 16 h. Supernatants were collected and analyzed for **(A)** IL-1β and **(B)** TNF-α release. Results are expressed as percentage of cytokine release relative to LPS-stimulated microglia (dark green bars). Data are means ± SEM of three independent experiments. *p < 0.05 and ***p < 0.001 versus LPS stimulation. One-way ANOVA followed by Sidak’s multiple comparison test. **(C)** At the end of 16 h incubation with E1 (1–250 μg/ml), cell viability was determined by SRB assay. Results are expressed as percentage of cell viability relative to control cells (dashed line). Data are presented as means ± SEM (*n* = 3 in triplicate). *p < 0.05 versus control cells. One-way ANOVA followed by Sidak’s multiple comparison test.

The effect of E1 on the release of the pro-inflammatory cytokines IL-1β and TNF-α on the initiation of microglia inflammatory response was examined. Cells were serum starved for 2 h, exposed for 1 h to increasing concentrations of the extract (1–100 μg/ml), and then stimulated with 100 ng/ml LPS to induce the inflammatory response. Unstimulated cells released low amounts of IL-1β and TNF-α which remained unchanged after treatment with the highest non-cytotoxic concentration of E1 (Figures 2A,B, white bars). In response to LPS, the release of IL-1β and TNF-α dramatically increased (Figures 2A,B, dark green bars) and E1 lowered it in a concentration-dependent manner. In particular, the extract completely inhibited the cytokine release starting from the concentration of 25 μg/ml (Figures 2A,B, light green bars). Considering that there was no effect on cell viability after treatment with the extract alone at concentrations ranging from 1-100 μg/ml (Figure 2C), these results indicate that the decrease in IL-1β and TNF-α release did not result from any cytotoxic effect.

**FIGURE 3:**
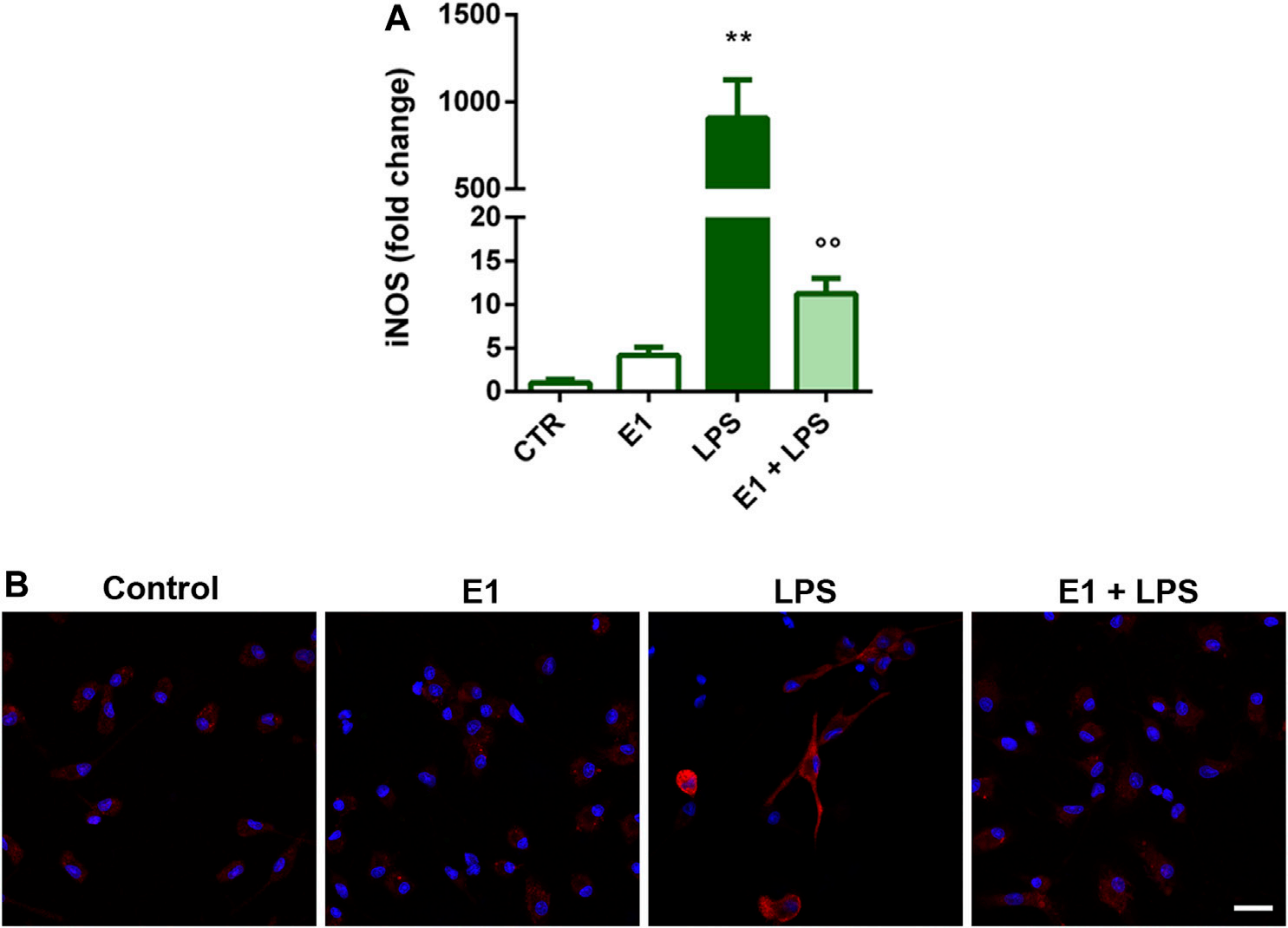
Effect of pre-treatment with Spirulina extract on iNOS gene and protein expression in LPS-stimulated cortical microglia. Microglia were cultured for 24 h in 10% serum-containing medium, which was replaced with serum-free medium before pre-treatment with 100 μg/mL E1 followed by stimulation with 100 ng/ml LPS. **(A)** iNOS mRNA levels were quantified by real-time PCR. Data are presented as means ± SEM (*n* = 3 in triplicate). **p < 0.01 compared to control cells; °°*p* < 0.01 versus LPS stimulation. One-way ANOVA followed by Sidak’s multiple comparison test. **(B)** Microglia were stained with anti-iNOS antibody (red) and nuclei with DAPI (blue). Experiments were performed three times and representative immunofluorescence images are shown. Scale bar, 20 μm.

Microglial activation by LPS is also accompanied by the increased expression of iNOS and the consequent nitric oxide production (Förstermann and Sessa, 2012). Therefore, we selected the extract concentration of 100 μg/ml to explore whether E1 pre-treatment could suppress the expression of iNOS upon LPS stimulation. The endotoxin induced gene and protein over-expression of iNOS, that was completely prevented by E1 (Figure 3), confirming the anti-inflammatory effect of E1 pre-treatment.

### Effect of Post-treatment With Spirulina Extract on Microglia Inflammatory Response

**FIGURE 4:**
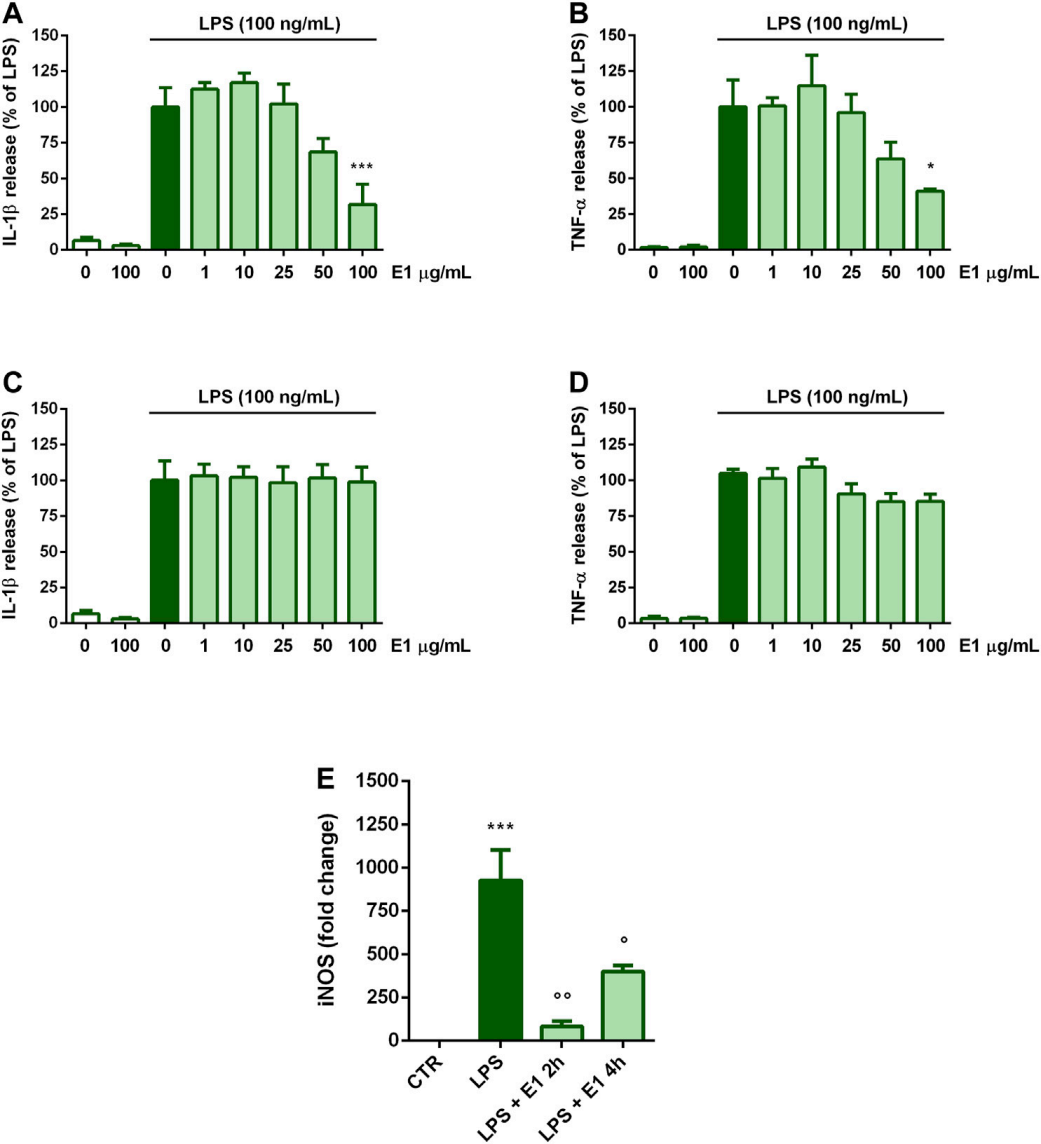
Effect of post-treatment with Spirulina extract on cytokine release and iNOS gene expression in LPS-stimulated cortical microglia. Microglia were cultured for 24 h in 10% serum-containing medium, which was replaced with serum-free medium before stimulation with 100 ng/ml LPS. **(A,B)** Two, or **(C,D)** 4 h later cells were treated with E1 (1–100 μg/ml). Supernatants were collected and analyzed for IL-1β and TNF-α release. Results are expressed as percentage of cytokine release relative to LPS-stimulated microglia (dark green bars). Data are means ± SEM of three independent experiments. *p < 0.05, **p < 0.01, and ***p < 0.001 versus LPS stimulation **(E)** One or 4 h after LPS stimulation cells were treated with 100 μg/ml E1 and iNOS mRNA levels were quantified by real-time PCR. Data are presented as means ± SEM (*n* = 3 in triplicate). ***p < 0.001 compared to control cells; °°*p* < 0.05 and °*p* < 0.01 versus LPS stimulation. One-way ANOVA followed by Sidak’s multiple comparison test.

Next, we explored whether E1 could decrease LPS-induced microglia inflammatory response when added after the initiation of inflammation. To test this, treatments with E1 started 2 or 4 h after LPS stimulation. When E1 was added to cells 2 h after LPS, the release of both cytokines decreased only at the concentration of 100 μg/ml (Figures 4A,B). Differently, E1 added 4 h post-LPS stimulation did not alter IL-1β and TNF-α release (Figures 4C,D).

Conversely, a significant down-regulation of iNOS gene expression was found when E1 was added 2 and 4 h after stimulation with LPS. However, although statistically significant, the effect on iNOS mRNA levels tended to diminish when cells were treated with the extract longer after the initiation of inflammation (i.e., 4 h after LPS stimulation) (Figure 4E).

To clarify the observed effect, the kinetic of IL-1β and TNF-α release was studied and, as shown in Table 2 and 3, the amount of both cytokines increased over the time. In particular, 2 h after LPS stimulation, levels of IL-1β and TNF-α released into the medium were 78.7 ± 15.5 pg/ml and 195.1 ± 51.2 pg/ml, respectively. Then, the release kept increasing and 16 h after LPS stimulation the levels of IL-1β and TNF-α were 805.3 ± 99.4 pg/ml and 945.3 ± 104.8 pg/ml, respectively. E1 added to cells 2 h after LPS stimulation significantly reduced the release of both cytokines; indeed, in the period 4–16 h after LPS stimulation, the levels of IL-1β and TNF-α released were in the range between 87.6 ± 21.6 pg/ml and 250.4 ± 61.3 pg/ml and 235.8 ± 32.1 pg/ml and 389.4 ± 91.7 pg/ml, respectively.

**TABLE 2:** Time course of IL-1β release (pg/ml) after LPS stimulation and effect of post-treatment with E1.

E1		Time (hours) after LPS stimulation				
2	4	0	2	4	8	16
**-**	**-**	2.4 ± 1.3	78.7 ± 15.5	257.1 ± 41.7	523.7 ± 79.7	805.3 ± 99.4
**+**	**-**	nd	nd	87.6 ± 21.6 * (−65.9 ± 11.5%)	157.6 ± 39.5 * (− 70.0 ± 13.5%)	250.4 ± 61.3 ** (− 68.9 ± 23.4%)
**-**	**+**	nd	nd	nd	487.3 ± 90.2	825.65 ± 101.32

E1 at the concentration of 100 μg/ml was added to cells 2 or 4 h after LPS stimulation (first and second column). Supernatant were collected at the indicated periods of time after LPS stimulation and analyzed for IL-1β release. Numbers in brackets denote the percentage change of IL-1β release compared to the corresponding treatment with LPS. Data are means ± SEM of three independent experiments.

*p < 0.05 and **p < 0.01 versus the corresponding LPS stimulation. Student’s *t* test. nd, not determined.

**TABLE 3:** Time course of TNF-α release (pg/ml) after LPS stimulation and effect of post-treatment with E1.

E1		Time (hours) after LPS stimulation				
2	4	0	2	4	8	16
-	-	2.4 ± 1.3	195.1 ± 51.2	489.1 ± 74.1	750.2 ± 87.7	945.3 ± 104.8
**+**	**-**	nd	nd	235.8 ± 32.1 * (−51.8 ± 17.3%)	397.2 ± 56.5 * (− 47.0 ± 23.7%)	389.4 ± 91.7 * (− 58.8 ± 18.8%)
**-**	**+**	nd	nd	nd	687.2 ± 140.9	926.5 ± 112.6

E1 at the concentration of 100 μg/ml was added to cells 2 or 4 h after LPS stimulation (first and second column). Supernatant were collected at the indicated periods of time after LPS stimulation and analyzed for TNF-α release. Numbers in brackets denote the percentage change of TNF-α release compared to the corresponding treatment with LPS. Data are means ± SEM of three independent experiments.

*p < 0.05 versus the corresponding LPS stimulation. Student’s *t* test. nd, not determined.

### Effect of Spirulina Extract on NF-κB Signaling in Microglia

**FIGURE 5:**
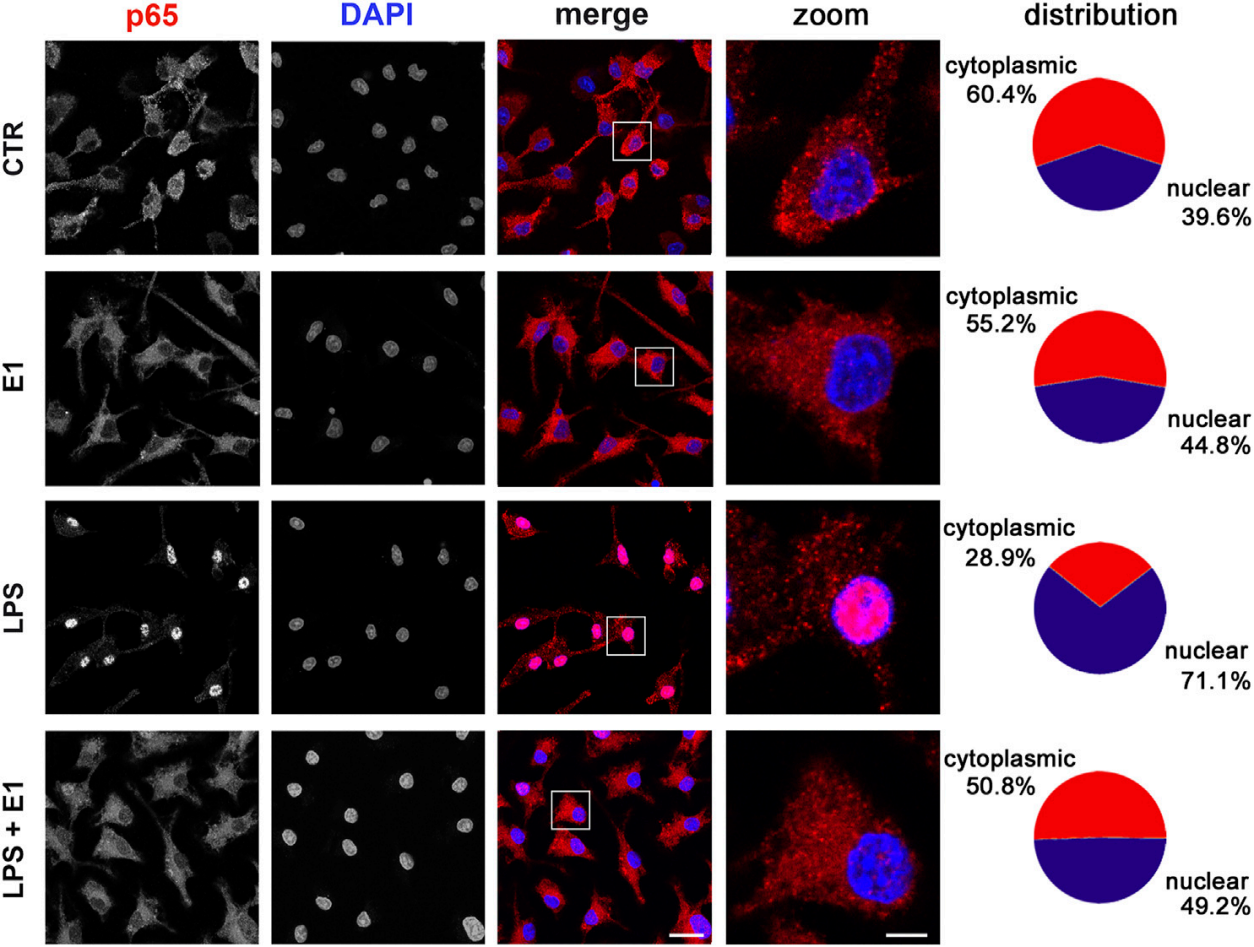
Effect of Spirulina extract on NF-κB activation in unstimulated and LPS-stimulated microglia. Cells were cultured for 24 h in 10% serum-containing medium, which was replaced with serum-free medium before pre-treatment with 100 μg/ml E1 followed by stimulation with 100 ng/ml LPS. Cells were then processed for NF-κB p65 immunostaining (red) and nuclei were counterstained with DAPI (blue). Experiments were performed 3 times and representative confocal images showing subcellular localization of p65 are shown. Scale bars in merged and zoomed images, 10 and 2 μm, respectively. The fluorescence intensity of cytoplasmic and nuclear p65 subunit was calculated using ImageJ software and results are presented as percentage of nuclear and cytoplasmic NF-κB p65 distribution in the fifth column. Data are mean from three independent experiments.

In the attempt to define the underlying molecular mechanisms by which the extract of Spirulina modulated microglia inflammatory response, we evaluated the activation of the transcription factor NF-κB, which is required for the induction of several cytokines and inflammatory enzymes in microglia and other immune cells (Zusso et al., 2017; Tedesco et al., 2018). To measure NF-κB activity, we monitored NF-κB p65 subunit movement from the cytoplasm to the nucleus. Under basal conditions and after E1 treatment p65 subunit was mainly distributed in the cytoplasm. As expected, LPS caused a significant translocation of the subunit to the nucleus, which was inhibited by pre-treatment with E1 (Figure 5).

### Effect of Spirulina Extract on Nrf2 Signaling in Microglia

**FIGURE 6:**
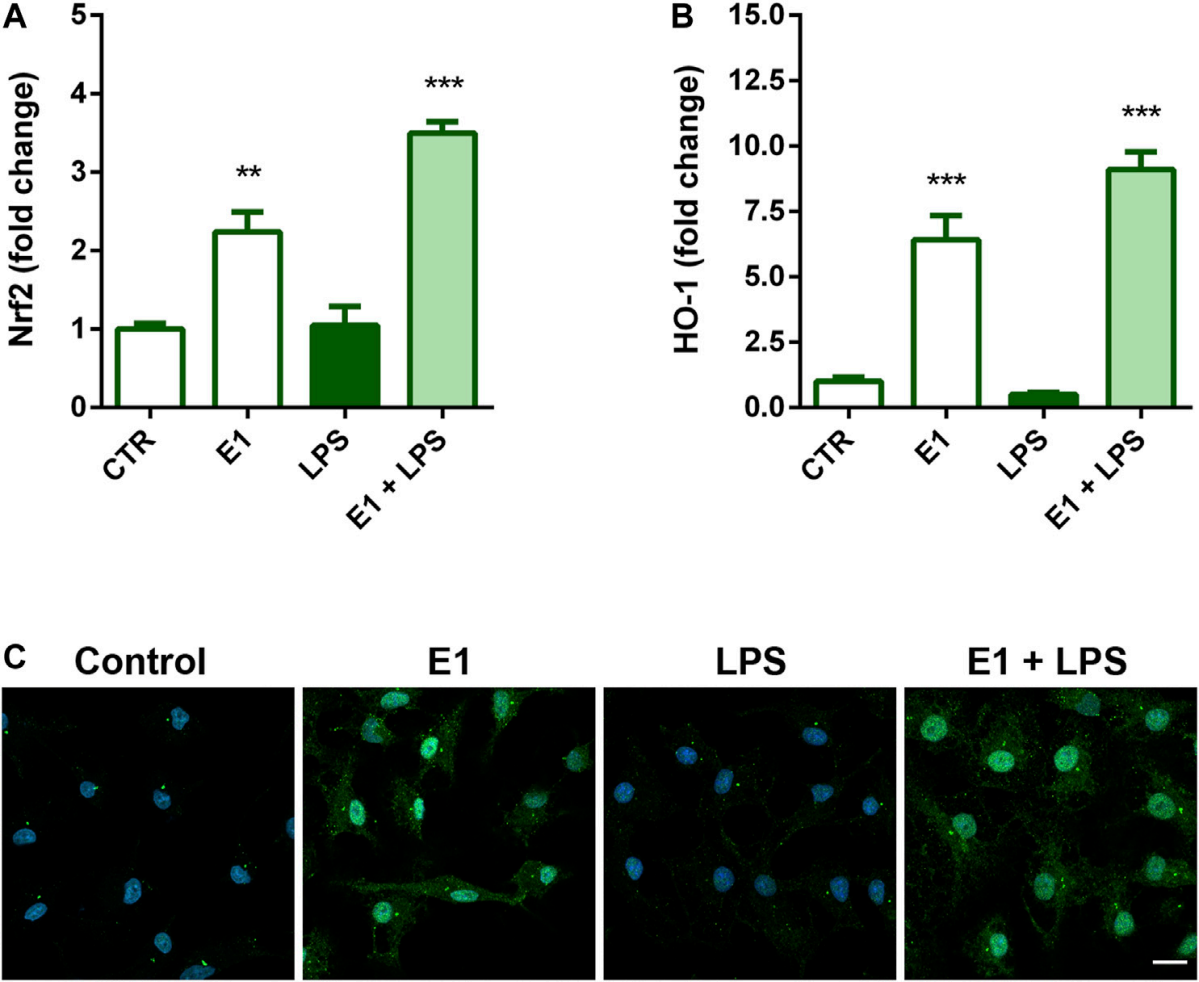
Effect of Spirulina extract on Nrf2 signaling in unstimulated and LPS-stimulated microglia. Cells were cultured for 24 h in 10% serum-containing medium, which was replaced with serum-free medium before pre-treatment with 100 μg/ml E1 for 1 h followed by stimulation with 100 ng/ml LPS. **(A)** Nrf2 and **(B)** HO-1 mRNA levels were quantified by real-time PCR. Data are presented as means ± SEM (*n* = 3 in triplicate). **p < 0.01 and ***p < 0.001 compared to control cells. One-way ANOVA followed by Sidak’s multiple comparison test. **(C)** Microglia were stained with anti-Nrf2 antibody (green) and nuclei with DAPI (blue). Experiments were performed three times and representative immunofluorescence images are shown. Scale bar, 10 μm.

Nrf2 signaling is the major regulatory system able to control the expression of antioxidant and detoxification enzymes and has also a role in mitigating inflammation (Kong et al., 2011; Kobayashi et al., 2016). Thus, we next examined the possibility that Nrf2 signaling might participate in the anti-inflammatory activity of E1. Pre-treatment with the extract increased gene expression of Nrf2 (Figure 6A) and its nuclear translocation (Figure 6C), as well as mRNA levels of HO-1 (Figure 6B), a phase II enzyme downstream of Nrf2, both in the absence and presence of LPS, suggesting a possible involvement of this signaling in the anti-inflammatory effect of the studied extract.

## Discussion

The present study investigated the properties of pre- and post-treatments with an acetone extract derived from the microalga Spirulina against inflammatory response in an *in vitro* model of LPS-induced neuroinflammation. Spirulina besides being an important source of nutrients, has been widely studied for its numerous beneficial effects in *in vitro* and *in vivo* models. For instance, Spirulina possesses anti-inflammatory, antioxidant, and neuroprotective properties, that can counteract chronic neurodegenerative disorders (Simpson and Oliver, 2020). The extract used in this study showed the presence of chlorophylls, pheophytins, and carotenoids, where β-carotene and zeaxanthin were the most abundant carotenoids. The present finding is in accordance with previous studies that reported the presence of these pigments in the microalga (Jaime et al., 2005; Hynstova et al., 2018). Chlorophylls and their magnesium-free degradation products, pheophytins, are considered protective agents against many chronic diseases, being endowed with antioxidant and antimutagenic/anticarcinogenic activity (Ferruzzi and Blakeslee, 2007; Wu et al., 2010; Martel et al., 2017). Furthermore, some studies have shown that chlorophylls and pheophytins exhibited promising anti-inflammatory activities (Subramoniam et al., 2012; Li et al., 2019). Carotenoids are lipid-soluble pigments produced by plants and some microorganisms. The consumption of carotenoids has been linked with various health conditions, including the prevention of neurodegenerative diseases, such as Alzheimer’s disease (Obulesu et al., 2011). Indeed, several studies have shown that carotenoids can accumulate in the CNS (Craft et al., 2004) and play a multitude of functions. The neuroprotective properties of carotenoids have been attributed to their action in the neural circuits by increasing neural efficiency or stabilizing membrane structures (Sujak et al., 1999). Furthermore, in the brain carotenoids inhibit lipid peroxidation, reduce oxidative damage by scavenging reactive oxygen species, and are anti-neuroinflammatory agents able to suppress various inflammatory pathways (Hammond, 2015; Cho et al., 2018). These observations support the hypothesis that, due to its composition, E1 could exert anti-neuroinflammatory activities. To test this, herein we studied the effect of E1 on microglia inflammatory response. Non-cytotoxic concentrations of E1 had preventive anti-inflammatory effects on LPS-stimulated microglia. These effects were associated with the suppression of IL-1β and TNF-α release, two of the most important and earliest cytokines produced during inflammation, whose sustained and high levels have been associated with neurodegeneration (Becher et al., 2017). Additionally, pre-treatment with E1 showed a significant effect on the inflammatory signaling also by decreasing the LPS-induced over-expression of iNOS, an important regulator of inflammation. These results confirm previous studies that have suggested the use of Spirulina as a natural product to prevent inflammatory diseases, based on the anti-inflammatory effect of organic or water extracts of the microalga (Chen et al., 2012; Ku et al., 2013; Pham et al., 2017). We recently showed very similar results obtained with the use of an acetone extract from the microalga *Euglena gracilis* on the same *in vitro* model of neuroinflammation, despite some differences in the composition of the two extracts. *Euglena gracilis* extract contained diadinoxanthin as the most abundant xanthophyll, followed by zeaxanthin, whereas β-carotene represented only 8% (Piovan et al., 2021). Conversely, the most abundant carotenoid present in the Spirulina extract was β-carotene, whereas the amount of zeaxanthin resulted comparable in the two extracts. These results suggest that zeaxanthin could play an essential role in the anti-inflammatory effect of Spirulina extract and will orient future studies aimed at the direct evaluation of single isolated components of the extract.

Of particular interest, in the present study, we showed that E1 not only had a preventive anti-neuroinflammatory effect, but it was also able to reduce the release of pro-inflammatory cytokines and the expression of inflammatory markers when administered to cells early after the initiation of the inflammatory response. However, this effect was restricted to the initial stages of neuroinflammation (i.e., 2 h after LPS stimulation), when pro-inflammatory cytokines were released into the medium but their amount did not reach maximal levels (Hide et al., 2000; Sanz and Di Virgilio, 2000). Furthermore, the inhibitory effect has been observed only after treatment of microglia with a high concentration of E1 (i.e., 100 μg/ml) and it progressively declined over the time, being lost in the advanced stage of neuroinflammation (i.e., 4 h after LPS stimulation), when the release of pro-inflammatory cytokines increased. These results clearly indicate the existence of a link between the timing of intervention and the entity of E1 effect on microglia activation, suggesting that E1, besides being a valid preventive option, may be of potential application in the early stages of inflammatory CNS diseases.

The expression of many cytokines, chemokines, receptors, and enzymes involved in inflammatory response is dependent on NF-κB pathway activation. In fact, an excessive or inappropriate activation of NF-κB has been associated with the development of inflammatory diseases and its inhibition can decrease the disease progression (Tak and Firestein, 2001; Lawrence, 2009; Liu et al., 2017). In the inactive form, NF-κB exists in the cytoplasm associated with the inhibitory proteins IκB. Inflammation leads to IκB phosphorylation and release of the heterodimer p50/p65, that, after translocation to the nucleus, binds to κB enhancer elements of target genes and induces the transcription of pro-inflammatory genes (Liu et al., 2017). Thus, monitoring NF-κB movement from the cytoplasm to the nucleus is a common method to measure NF-κB activity. In microglia cells, NF-κB p65 nuclear translocation induced by LPS was significantly attenuated by pre-treatment with E1, suggesting that the inhibition of NF-κB activation could be one of the potential anti-inflammatory mechanisms of the studied extract. In previous studies the anti-inflammatory effect of carotenoids has been ascribed to the inhibition of NF-κB activity, through a mechanism that involved the inhibition of DNA-binding activity of p65 (Palozza et al., 2003; Linnewiel-Hermoni et al., 2014; Li et al., 2019). Therefore, we cannot exclude the possibility that E1 components can act by directly interacting with NF-κB.

Oxidative stress, associated with increased levels of reactive species and a decrease in the antioxidant systems, has been implicated in the development and maintenance of inflammation and progression of neurodegenerative diseases. In this context, Nrf2 signaling, in addition to control intracellular redox homeostasis, has anti-inflammatory properties and has emerged as a therapeutic target in neurodegenerative conditions (Brandes and Gray, 2020). This signaling acts as an environmental sensor that allows cells to monitor for the presence of insults. Once activated, Nrf2 signaling leads the subsequent transcription of genes that are involved in antioxidant and anti-inflammatory responses. Thus, stimulation of Nrf2 appears a promising method for reducing the level of neuroinflammation and neurodegeneration. In our experimental conditions, E1 alone increased the expression of Nrf2 and that of its downstream gene HO-1, a potent anti-inflammatory target, suggesting that the induction of an antioxidant response might contribute, at least in part, to the anti-inflammatory properties of the extract components. However, more studies are necessary to verify a possible direct interaction of the extract components with Nrf2, as already showed for some carotenoids and their derivatives that can interact with Keap1, the negative regulator of Nrf2, by changing its physical properties (Kaulmann and Bohn, 2014). However, in this regard, it should be noted that, despite Nrf2 is considered a key regulator of inflammatory processes (Ahmed et al., 2017), based on the existence of a complex interaction between oxidative stress and inflammation, we recently showed that the anti-inflammatory effect of the acetone extract of *Euglena gracilis* is Nrf2 independent (Piovan et al., 2021). Similarly, the anti-inflammatory effect of E1 could be independent of the activation of Nrf2.

Taken together, our data indicate that the acetone extract from Spirulina can suppress the activation of NF-κB and induce the activation of Nrf2/HO-1, two intracellular signaling pathways that could act independently or in complementary manner. Clearly, additional studies using pathway specific inhibitors must be considered to elucidate the precise anti-inflammatory mechanism and explore other potential mechanisms. However, the results of this study represent promising evidence to support future *in vivo* studies to test the effect of pre-treatment (i.e., prophylactic effect) and post-treatment with the acetone extract from Spirulina.

## Data Availability

The original contributions presented in the study are included in the article/supplementary files, further inquiries can be directed to the corresponding author.
